# A245 DETERMINING THE PREVALENCE OF DELAYED GASTRIC EMPTYING IN PEDIATRIC PATIENTS WITH CONSTIPATION USING NUCLEAR SCINTIGRAPHY

**DOI:** 10.1093/jcag/gwab049.244

**Published:** 2022-02-21

**Authors:** A Rogalsky, M Gould, R Vali, P Marcon

**Affiliations:** 1 University of Toronto Temerty Faculty of Medicine, Toronto, ON, Canada; 2 The Hospital for Sick Children, Division of Gastroenterology, Hepatology and Nutrition, Toronto, ON, Canada; 3 The Hospital for Sick Children, Division of Nuclear Medicine, Department of Diagnostic Imaging, Toronto, ON, Canada

## Abstract

**Background:**

Gastrointestinal motility is coordinated by complex neurohormonal circuits existing between different segments of the gastrointestinal tract. It has been hypothesized that one of these pathways, the “cologastric brake,” acts as a negative feedback system to inhibit gastric motility when there is loading of stool within the colon in conditions such as constipation.

**Aims:**

To assess gastric emptying in pediatric patients presenting with constipation using whole-gut nuclear scintigraphy. We hypothesize that children with constipation are likely to exhibit delayed gastric emptying due to neurohormonal pathways such as the cologastric brake.

**Methods:**

A retrospective review of all patients under 18 years of age who underwent a whole-gut nuclear scintigraphy study for the work-up of constipation at The Hospital for Sick Children between January 2014 and May 2021 was completed. Demographic and clinical data at the time of presentation was summarized. The proportion of patients with delayed gastric emptying and delayed small and large bowel transit were calculated. Exploratory univariable analyses were performed to identify clinical predictors of delayed gastric emptying.

**Results:**

This study identified 140 patients meeting inclusion criteria of whom 72 were male (51.4%), (mean_age_ = 10.04 ± 4.79). The prevalence of patients with delayed gastric emptying was 61.9% (n = 86). Despite this, only 5.0% (n = 7) and 21.8% (n = 29) had delayed small and large bowel transit respectively. Children with delayed gastric emptying were significantly younger (mean_age_ = 9.15 ± 4.60) than those with normal gastric emptying (mean_age_ = 11.53 ± 4.79), *p* = .01. A chi-square test of independence showed that there was no significant relationship between use of a promotility agent and delayed gastric emptying ( *p* = .48). There was also no significant association between the presence of upper gastrointestinal symptoms and delayed gastric emptying ( *p* = .79).

**Conclusions:**

We found that children with constipation have a high prevalence of delayed gastric emptying. However, only a small proportion of these patients have delayed bowel transit. This supports the idea that delayed gastric emptying in children with constipation is likely not due to intrinsic neuromuscular defects of the gut but rather, secondary to a neurohormonal “cologastric brake.” This is important information for clinicians as it suggests that a key step in managing delayed gastric emptying in this scenario is to treat patients’ constipation as opposed to initiating therapies, such as prokinetic agents, which attempt to directly improve gastric emptying.

**Funding Agencies:**

None

